# Supplementary material for: Implementation of a Canine Ergonomic Abdominal Simulator for Training Basic Laparoscopic Skills in Veterinarians
Source: Animals (Basel). 2023 Mar 23;13(7):1140. doi: 10.3390/ani13071140 (PMC10093257; doi:10.3390/ani13071140)
Supplement: Supplementary file 1 [file animals-13-01140-s001.zip › Annex S1. Basic training plan exercises..pdf]

Annex S1. Basic training plan exercises.

Exercise	Description	Requirements
**Transfer of objects with the right hand**	Move six objects from the left side of the board to the right side with the right hand, and finally, reverse the exercise. If an object falls, it is not penalized, but at the end of the exercise, it is rearranged to its initial place and continued.	Perform two consecutive repetitions in a timeless than or equal to 48 seconds
**Transfer of objects by the left hand**	Move six objects from the left side of the board to the right side with the right hand, and finally, reverse the exercise. If an object falls, it is not penalized, but at the end of the exercise, it is rearranged to its initial place and continued.	Perform two consecutive repetitions in a timeless than or equal to 48 seconds
**Transfer of objects with the passage between both hands.**	Move six objects from the left side of the board to the right side with the right hand, and finally, reverse the exercise. If an object falls, it is not penalized, but at the end of the exercise, it is rearranged to its initial place and continued.	Perform two consecutive repetitions in a timeless than or equal to 48 seconds
**Gauze cutting with curved scissors in the right hand**	Move six objects from the left side of the board to the right side with the right hand, and finally, reverse the exercise. If an object falls, it is not penalized, but at the end of the exercise, it is rearranged to its initial place and continued.	Perform two consecutive repetitions in a timeless than or equal to 98 seconds
**Gauze cutting with curved scissors in the left hand**	Cut half a circle from the left side of non-woven gauze with your left hand, making clean cuts on the demarcated line. The size of the non-woven gauze sponge (cotton-polyester) was 10 cm x 15 cm, double fold, with a circular pattern in the center of 4 cm in diameter, 3 mm in line thickness, and suspended between alligator clips.	Perform two consecutive repetitions in a timeless than or equal to 98 seconds
**Interrupted intracorporeal suture task**	Perform a simple suture with four intracorporeal knots through two punctiform marks in a Penrose. A ¾" and 5 cm long slotted Penrose drain was used. The guide points were 5 mm apart. A 2-0 silicone braided black silk was used, and a CR-30 needle with a length of 15 cm.	Perform two consecutive repetitions in a timeless than or equal to 75 seconds

**Continuous intracorporeal suture task.**	Perform a running suture, knotted at both ends, passing the needle through 8 punctate marks on a Penrose. A ¾" and 5 cm long slotted Penrose drain was used. The guide points were 5 mm apart. A 2-0 silicone braided black silk was used, and a CR-30 needle with a length of 15 cm.	Perform two consecutive repetitions in a timeless than or equal to 240 seconds
