# Supplementary material for: Implementation of a Canine Ergonomic Abdominal Simulator for Training Basic Laparoscopic Skills in Veterinarians
Source: Animals (Basel). 2023 Mar 23;13(7):1140. doi: 10.3390/ani13071140 (PMC10093257; doi:10.3390/ani13071140)
Supplement: Supplementary file 1 [file animals-13-01140-s001.zip › Annex S2. Face and content validity survey of the CVLTS..pdf]

Annex S2. Face and content validity survey of the CVLTS.

Date: _MM_/_DD_/_YY_

Participant name: ______________________________ participant code: ___________

Satisfaction survey of the CVLTS					
	Score				
**Questionnaire about CVLTS items ***	1	2	3	4	5
	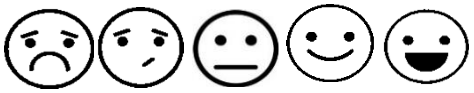				
**It is realistic, didactic, and of a suitable size for training in basic laparoscopic skills**					
**It has a clear, friendly, and colorful image quality**					
**Range of exercises**					
**It is helpful for the training of veterinary students**					
**It is helpful for the training of veterinary surgeons**					
**It would help improve my laparoscopic skills and apply them to my patients**					
**Do you consider the inclusion of the CVLTS useful in laparoscopy training programs for veterinary students before practice in the operating room?**					
**If you have already practiced on other simulators, would you prefer to use the CVLTS instead?**					
**Suggestions and comments:**					
***Assessment (1: Very negative; 5: Very positive)**					

Survey to determine the apparent validity and content, product of scientific article. Author: Usón et al., 2014.[10]
